# Atmospheric Pressure Plasma: A High-Performance Tool for the Efficient Removal of Biofilms

**DOI:** 10.1371/journal.pone.0042539

**Published:** 2012-08-06

**Authors:** Katja Fricke, Ina Koban, Helena Tresp, Lukasz Jablonowski, Karsten Schröder, Axel Kramer, Klaus-Dieter Weltmann, Thomas von Woedtke, Thomas Kocher

**Affiliations:** 1 Leibniz Institute for Plasma Science and Technology e.V. (INP Greifswald), Greifswald, Germany; 2 Unit of Periodontology, Dental School, University of Greifswald, Greifswald, Germany; 3 Centre for Innovation Competence Plasmatis, Greifswald, Germany; 4 Institute for Hygiene and Environmental Medicine, University of Greifswald, Greifswald, Germany; Consejo Superior de Investigaciones Cientificas, Spain

## Abstract

**Introduction:**

The medical use of non-thermal physical plasmas is intensively investigated for sterilization and surface modification of biomedical materials. A further promising application is the removal or etching of organic substances, e.g., biofilms, from surfaces, because remnants of biofilms after conventional cleaning procedures are capable to entertain inflammatory processes in the adjacent tissues. In general, contamination of surfaces by micro-organisms is a major source of problems in health care. Especially biofilms are the most common type of microbial growth in the human body and therefore, the complete removal of pathogens is mandatory for the prevention of inflammatory infiltrate. Physical plasmas offer a huge potential to inactivate micro-organisms and to remove organic materials through plasma-generated highly reactive agents.

**Method:**

In this study a *Candida albicans* biofilm, formed on polystyrene (PS) wafers, as a prototypic biofilm was used to verify the etching capability of the atmospheric pressure plasma jet operating with two different process gases (argon and argon/oxygen mixture). The capability of plasma-assisted biofilm removal was assessed by microscopic imaging.

**Results:**

The *Candida albicans* biofilm, with a thickness of 10 to 20 µm, was removed within 300 s plasma treatment when oxygen was added to the argon gas discharge, whereas argon plasma alone was practically not sufficient in biofilm removal. The impact of plasma etching on biofilms is localized due to the limited presence of reactive plasma species validated by optical emission spectroscopy.

## Introduction

Physical plasmas are fully or partially ionized gases which are generated by supplying energy to gaseous medium leading to dissociation of molecular bonds and ionization reactions. Hence, plasma consists of positively and negatively charged ions, electrons as well as neutral atoms and molecules (e.g. radicals) [1]. Furthermore, depending on the ionized gas, ultraviolet radiation can be emitted. Plasma can be categorized in high temperature or low temperature plasmas. The latter group of plasmas is subject of the present paper. In low temperature plasmas noble gases, such as argon and helium, and chemically active gases (e.g. oxygen and nitrogen) are commonly used. Moreover, low temperature plasmas are classified by the temperature of all species (electrons, ions, and neutral species) in thermal and non-thermal plasmas [1]. Owing to its low neutral gas temperature (near or around room temperature) non-thermal plasmas may offer new biomedical applications such as bio-decontamination or sterilization of heat sensitive materials (e.g. polymers) and modification of surfaces for subsequent medical applications [2]. Furthermore, due to the moderate gas temperature of non-thermal plasmas and, above all, the possibility of their generation at atmospheric pressure conditions, the application of these plasmas on living tissues is possible [3]. The effect of non-thermal plasmas on the inactivation of micro-organisms has been extensively studied over the past decade [4] concluding that effective inactivation of micro-organisms is based on plasma-generated highly reactive agents including UV photons, oxygen species (O_2_^−^, O_3_, O), charged particles as well as electric fields [5], [6], [7], [8]. Inactivation of microbial pathogens is important, but a further challenging task is the complete removal of killed micro-organisms and their organic remnants by physical plasmas. Plasma appears to be a promising technique to eliminate organic material from surfaces without having toxic residue effects [9] and without damaging the underlying surface.

Biofilms are a serious medical problem [10], [11]. They consist of a complex system of micro-organisms embedded within a self-produced extracellular matrix [12]. The extracellular matrix stabilizes the biofilm architecture, embeds essential materials like nutrients from the surrounding environment, and provides a certain degree of resistance against environmental threats, antimicrobial agents, and the host immune response [13]. Pathogenic biofilms are involved in 65% or more of nosocomial infections [14]. For instance, *C. albicans* is considered as the most prevalent fungal biofilm-forming pathogen which causes life-threatening infections by colonizing polymers used for medical devices, such as dental material, stents, prostheses, implants, and catheters [13]. Therefore, *C. albicans* biofilm was used as a prototypic biofilm in this study. To minimize the risk of infections, surfaces can be cleaned and disinfected with different methods including ultrasound, ionizing radiation, and antimicrobial agents [15]. In general, for the removal of biofilms the mechanical procedure is the method of choice, because antimicrobial agents cannot penetrate into and inactivate biofilms [11], [16]. But among the conventional techniques, research efforts have led to the development of alternative methods for the elimination of micro-organisms by applying non-thermal plasmas. The possibility to remove biological contaminants using physical plasmas was first mentioned by Whittaker *et al.*, which investigated the application of low-pressure plasma for cleaning of endodontic files [17]. Since then, only few scientists have done investigations in the field of plasma-assisted etching of biological substances. Baxter *et al.* studied the removal of prions from surgical instruments exposed for one hour to Ar/O_2_ low-pressure plasma [18]. Rossi *et al.* reported an etching rate of 20 nm/s for a protein layer of bovine albumin which was exposed to Ar/O_2_ plasma at low-pressure [19]. However, these studies were focused on using vacuum plasma technologies to eliminate proteins. In recent years, a lot of effort has been put into the development of non-thermal plasma devices operating at atmospheric pressure. But so far, little is reported on the application of atmospheric pressure plasma for etching of organic substances. In particular, the capability of atmospheric pressure plasma for the elimination of complex biological systems (e.g. biofilms) is not extensively studied, yet. Ermolaeva *et al.* investigated the bactericidal effect of several minutes of Ar plasma on *Pseudomonas aeruginosa* biofilms [20]. Rupf *et al.* studied the removal of dental biofilms by means of an atmospheric pressure plasma jet operating with helium as well as a combination of plasma and water spray [21].

In preliminary studies the impact of plasma-generated species, provided by the atmospheric pressure plasma jet, on synthetic polymers was already investigated to obtain detailed information on the plasma-initiated etching mechanism (e.g. influence of the applied process gas and of the operating distance on the etching efficacy) [22]. Furthermore, synthetic polymers were chosen since the elemental compositions of these polymers are in some way similar to those of cell constituents of micro-organisms. Hence, the studied polymers can be considered as model constituents for cell compounds whose elemental composition mainly include C, H, O, N, and P. For instance, the cell wall of *C. albicans* is predominantly composed of 80–90% of carbohydrates, 6–25% of proteins, and 1–7% of lipids [23], whereas the biofilm matrix of *C. albicans* mainly contains polysaccharides [24]. Therefore, the present study is focused on the transition of the investigations obtained on abiotic surfaces to living micro-organisms organized in a biofilm to determine the efficacy of the atmospheric pressure plasma jet in etching of micro-organisms. Besides, based on its similar and, most important, its well defined chemical composition, synthetic polymers can be easily analyzed by surface techniques under defined conditions compared to complex microbial systems. Thus, the etching rates of these materials will be compared to etching rates estimated for the biofilm. Furthermore, the use of synthetic polymers allows a more detailed look into the mechanistic pathway of substance removal.

This contribution is focused on analyzing: 1. The etching efficacy of argon (Ar) and argon/oxygen (Ar/O_2_) atmospheric pressure plasma jet on 7-day old *C. albicans* biofilms. 2. Estimation of etching rates by the determination of the biofilm thickness. 3. The dimension of the plasma-influenced area, by using the synthetic polymer poly(ether ether ketone) (PEEK) as model for the biofilm, as it features similar etching rates. 4. The influence of plasma-generated species on the etching process by performing optical emission spectroscopy (OES).

## Results and Discussion

**Figure 1:**
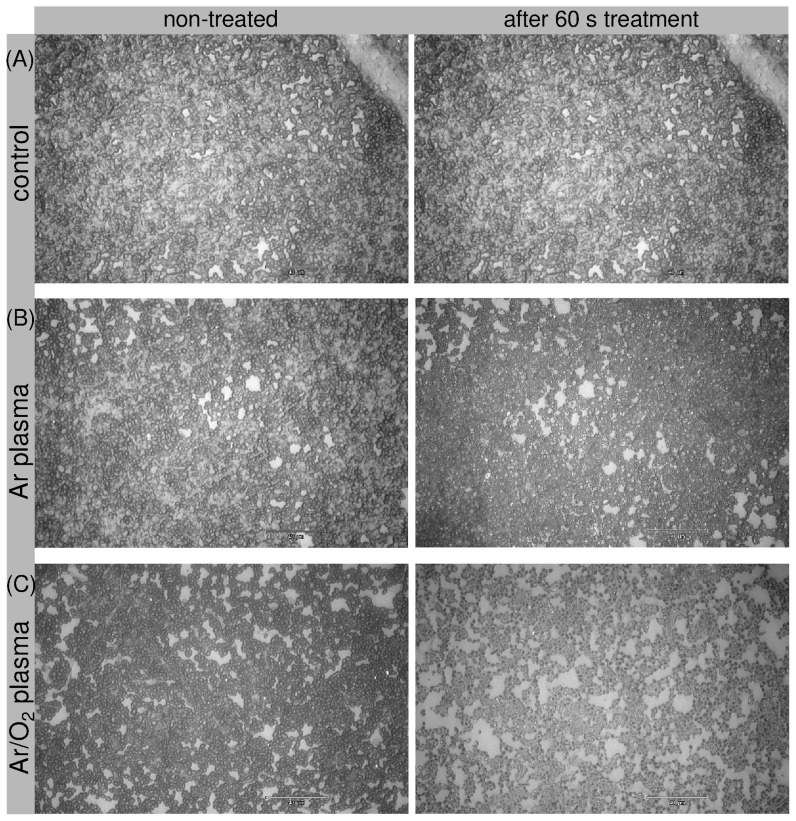
Influence of different gas discharge plasmas on 7-day old *Candida albicans* biofilms grown on polystyrene wafer. The samples were washed and dried by air flow before plasma treatment. The microscopic images were taken with a magnification of 200 at the same sample position before (left column) and after plasma treatment (right column) A: The control sample was only exposed to the Ar/O_2_ gas flow for 60 s without plasma ignition. B: The biofilm sample was exposed to 5 slm Ar plasma. C: The biofilm sample was exposed to plasma composed of a gas mixture of 5 slm Ar and 0.05 slm O_2_ (total admixture of 1% O_2_).

**Figure 2:**
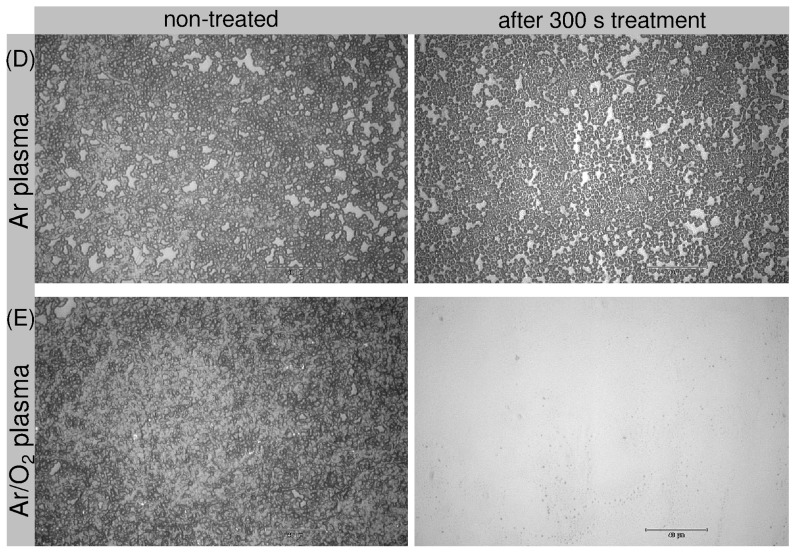
Plasma-dependent removal of 7-day old *Candida albicans* biofilms grown on polystyrene wafer. exposed to: (D) Ar gas discharge plasma (5 slm Ar) and (E) Ar/O_2_ gas discharge plasma (5 slm Ar+0.05 slm O_2_) for 300 s.

**Figure 3:**
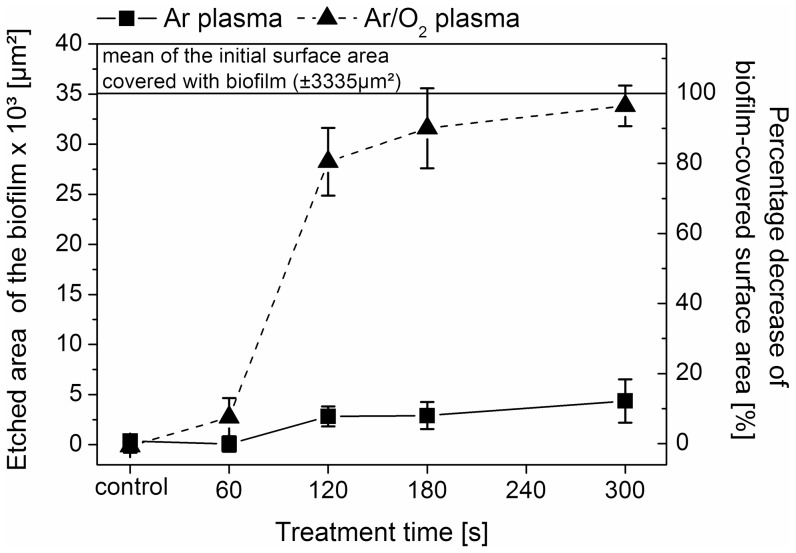
Comparison between Ar plasma 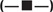 and Ar/O_2_ plasma 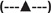 on etched surface area of 7-day old *Candida albicans* biofilms depending on the plasma treatment time (n = 5; mean ± SD). The biofilm-covered surface area was measured before and after plasma treatment. The control represents samples exposed to the gas flow without plasma ignition for 60 and 300 s. The initial surface area of the biofilm was 35171±3335 µm^2^ (n = 60). The right y-axis represents the percentage decrease of the biofilm-covered surface area.

The investigations demonstrate the removal or etching of biofilms by means of an atmospheric pressure plasma jet and extend our previous data, in which the efficacy of the plasma jet in killing, but not in removal of micro-organisms, was shown [25], [26]. Figure 1 shows representative images of *C. albicans* biofilms before and after 60 s (A) Ar/O_2_ gas flow (control), (B) Ar plasma, and (C) Ar/O_2_ plasma exposure. Initial removal - apparent as bright spots representing the PS subsurface - of *C. albicans* biofilm was observed after 60 s Ar plasma treatment (Fig. 1 (B)). Distinct biofilm removal, however, could be observed only with Ar/O_2_ plasma (Fig. 1 (C)). Figure 2 depicts a comparison of non-treated biofilm and biofilm exposed to (D) Ar and (E) Ar/O_2_ gas discharge plasma for 300 s. As can be seen in Fig. 2 D, longer Ar plasma exposure time did not result in substantial etching of the biofilm. In contrast, an almost complete removal of biofilm was obtained after Ar/O_2_ plasma treatment (Fig. 2 (E)). Consequently, to obtain increased biofilm removal, long time exposure was required. All control samples exposed to non-ionized Ar and Ar/O_2_ gases for 60 s and 300 s showed no biofilm etching. Hence, the process gas alone did not result in biofilm removal. To estimate the etching efficacy of the plasma jet, the biofilm-covered surface area of each sample was calculated before and after plasma exposure. The results of plasma-etched surface areas, i.e. the difference of the biofilm-covered area before and after exposure to gas discharge plasma, depending on treatment times and process gases, are displayed in Fig. 3. As previously noted, even longer treatment times with Ar plasma did not significantly increase the etched biofilm surface area. After 300 s Ar plasma exposure a maximum surface area of 5000 µm^2^ of initial 35171 µm^2^ (mean of the untreated biofilm-covered surface area) was etched. Compared to Ar plasma treatment, the admixture of oxygen increased the biofilm removal considerably, especially for treatment times above 60 s. Further shown in Fig. 3, is the percentage decrease of the biofilm (right y-axis). A reduction in biofilm-covered surface area of only 12% was attained with 300 s Ar plasma. In contrast, already after 180 s Ar/O_2_ plasma exposure, approximately 95% of the biofilm was etched. Consequently, the admixture of oxygen is crucial to obtain a sufficient efficacy in biofilm removal.

**Figure 4:**
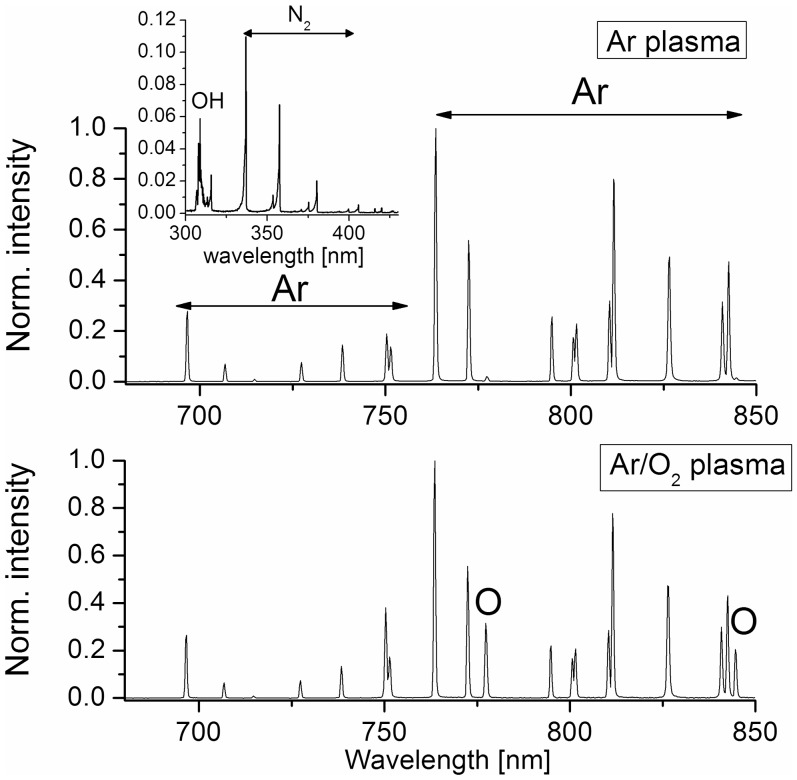
Intensity of excited plasma-generated species in the Ar and Ar/O_2_ gas discharge. Overview optical emission spectra in the visible range (680–850 nm) of Ar plasma (5 slm Ar) and Ar/O_2_ plasma (5slm Ar+0.05 slm O_2_) measured at a distance of 7 mm to the jet-nozzle by means of a dual channel fiber optical spectrometer (Avantes AvaSpec 2048-2-USB2). The spectra were relative calibrated, normalized to the exposure time, and analyzed using the software Spectrum Analyzer. In the upper spectrum excited species generated in the Ar gas discharge are shown, in particular atomic Ar in the range of 680–850 nm. The inset figure shows the emission spectrum of Ar plasma in the UV range (300–430 nm) which exhibits emission lines of OH at 309 nm and of the 2nd positive system of N_2_ at 337–391 nm. The lower spectrum exhibits excited species generated in Ar/O_2_ plasma which is dominated by emission lines of atomic Ar and additional emission lines of atomic O at 777.4 nm and 844.6 nm. Not shown is the emission spectrum in the UV range due to the absence of emission lines.

To gain further insight into the mechanism of biofilm etching and to identify reactive plasma species that are involved in the surface effects induced by plasma exposure, the plasma was characterized by performing optical emission spectroscopy. The spectral characteristics in the ultraviolet/visible (UV/VIS) range of Ar and Ar/O_2_ plasma are plotted in Fig. 4. The emission spectrum of the Ar gas discharge was mainly dominated by atomic lines of Ar between 670 and 850 nm in the visible range. Furthermore, emission lines of OH at 309 nm and of the second positive system of molecular nitrogen N_2_ at 337 nm were identified in the UV range (240–400 nm). The OH radical was probably formed from water vapors present in the ambient air [27]. In contrast, the optical emission spectrum of Ar/O_2_ plasma showed additional emission lines of high intensity of atomic oxygen at 777.4 nm and 844.6 nm in the visible region which were caused by dissociative excitation and direct excitation processes. Neither emission lines of OH nor of N_2_ were observed in the UV range of Ar/O_2_ plasma. Consequently, the admixture of oxygen resulted in collisional quenching of N_2_ and OH lines [28] which suppressed the excitation and production mechanisms. Since the previous results indicated a reduced etching efficacy by using Ar plasma, it is most likely that UV radiation had a little effect on biofilm etching. Furthermore, the comparison of the ratio of the intensity of the emission lines of O (I_O_) at 844.6 nm and Ar (I_Ar_) at 750.4 nm of both gas discharges revealed an I_O_/I_Ar_ ratio of 0.6 for Ar/O_2_ plasma and an I_O_/I_Ar_ ratio of 0.05 for Ar plasma. Hence, the portion of oxygen in the spectrum of Ar/O_2_ plasma is remarkable higher. Deduced from these results it can be assumed that reactive oxygen species played a major role for biofilm removal and hence, that plasma-assisted etching was mainly a chemically driven process.

In preliminary studies synthetic polymers were investigated with respect to the capability of the applied plasma jet to etch hydrocarbon based aliphatic and aromatic polymers [22]. Especially, the impact of different process gases (argon and different argon/oxygen mixtures) and the influence of the chemical structure on etching rates were examined. Briefly, the etching rates of aliphatic polymers (polyethylene, polypropylene, and poly(methyl methacrylate)) varied between *R* = 180 nm/s and *R* = 300 nm/s, whereas the etching rates of aromatic polymers (polystyrene, polycarbonate, and poly(ether ether ketone)) were comparatively lower, ranging from *R* = 50 nm/s for poly(ether ether ketone) up to *R* = 150 nm/s for polycarbonate. Consequently, the etching rate depends not only on the process gas, but also on the chemical structure of the material to be etched.

The calculation of the etching rate requires the measurement of the thickness of the *C. albicans* biofilm, which equaled the distance between the lowest position (surface of the PS wafer) and the highest position (top of the biofilm). Since the best result in biofilm removal was achieved using Ar/O_2_ gas discharge plasma with treatment times of 300 s, the etching rates were estimated only for this exposure time. Hence, before plasma treatment, the biofilm thickness was measured revealing a minimum biofilm thickness of approximately 10 µm and a maximum biofilm thickness of approximately 20 µm (these data are obtained from 5 biofilm-covered PS wafers; n = 3 on each sample). These variations in thicknesses were due to the inhomogeneous biofilm formation. Consequently, biofilm etching rates of *R* = 33 nm/s (for the biofilm thickness of 10 µm) to *R* = 67 nm/s (for the biofilm thickness of 20 µm) with Ar/O_2_ plasma could be computed.

Since the investigations on biofilms revealed some drawbacks, such as the restricted thickness of biofilms to several micrometers, the variation of the thickness due to its inhomogeneous growth, and their sensitivity towards some surface analysis techniques, the polymer PEEK, as a substitute for biofilm, was used to overcome these limitations. Furthermore, the use of a model ensured consistent and reproducible conditions for a detailed analysis of the etching process. Additionally, PEEK was chosen since its etching rate is similar to the etching rates of the biofilm obtained in this study [22].

**Figure 5:**
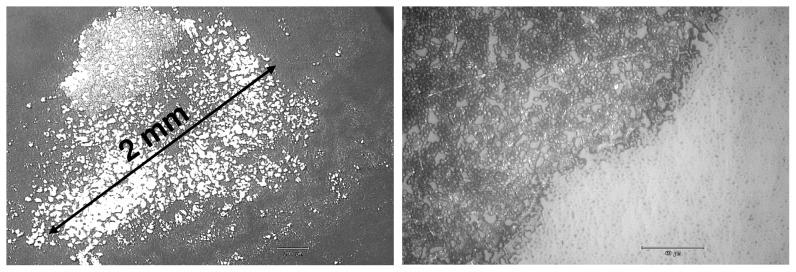
Extent of the plasma-etched surface area after 180 s Ar/O_2_ plasma exposure. Images taken at 50-fold magnification on the left and at 200-fold magnification on the right. Left: The dark area represents the densely packed biofilm whereas the bright areas display the PS wafer surface. An etched area of 2 mm in diameter was measured. Right: Sharp boundary between the plasma-affected area and the still present biofilm.

**Figure 6:**
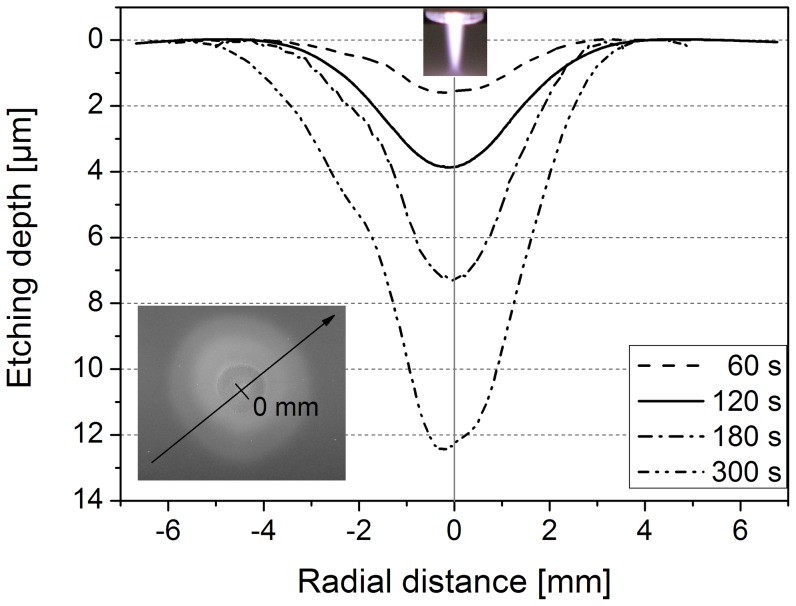
Effectively plasma-etched surface of poly(ether ether ketone) (PEEK) dependent on the treatment time. Radial surface profile of PEEK exposed to Ar/O_2_ gas discharge plasma (5 slm Ar+0.05 slm O_2_) for 60, 120, 180, and 300 s at a jet-nozzle to substrate distance of 7 mm recorded by a stylus surface profiler. ‘0’ represents the position of the localized plasma treatment. Inset figure: depiction of the surface profile measurement on PEEK. In the centre of the polymer (position 0 mm) the etched crater can be observed.

**Figure 7:**
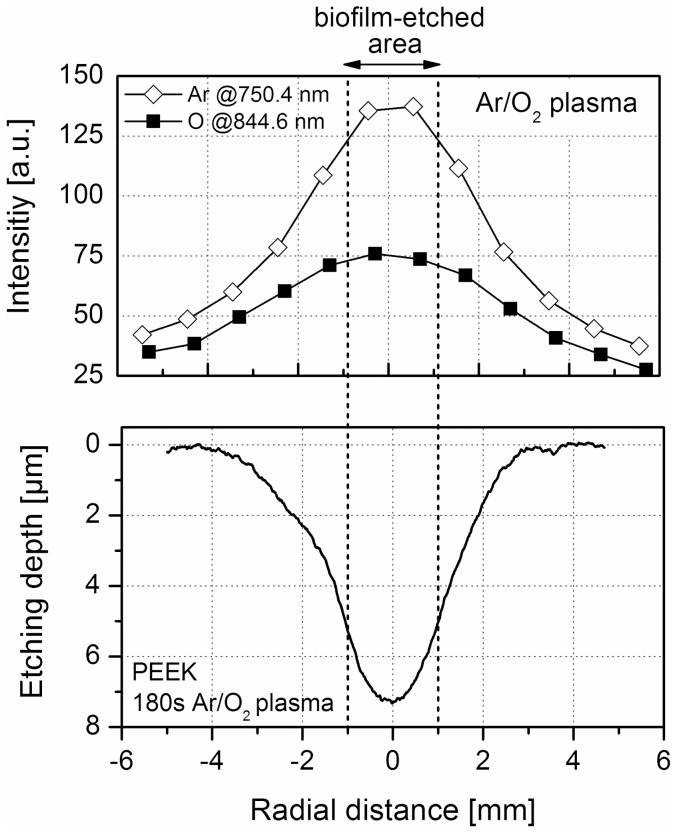
Radial distribution of plasma-generated species in the Ar/O_2_ gas discharge plasma and the direct relation to the radial etching profile of poly(ether ether ketone) (PEEK). The upper graph shows the intensity of atomic Ar at 750.4 nm and atomic O at 844.6 nm depending on the radial distance recorded end-on at a distance of 7 mm to the jet-nozzle by means of a dual channel fiber optical spectrometer (Avantes AvaSpec 2048-2-USB2). The spectra were relative calibrated, normalized to the exposure time, and analyzed using the software Spectrum Analyzer. ‘0’ represents the position of the atmospheric pressure plasma jet. In the lower figure the radial etching profile of PEEK after 180 s Ar/O_2_ plasma exposure is displayed. The surface profile was recorded by means of a stylus surface profiler (Dektak 3ST, Veeco, USA). The dashed lines mark the extent of the biofilm-etched surface (see Fig. 5).

An important aspect of biofilm removal was the localized effect of the plasma treatment. Figure 5 exemplarily shows that the effective biofilm removal was restricted to a few millimeters after 180 s Ar/O_2_ plasma exposure. The dark regions represent the densely packed *C. albicans* biofilm, whereas the bright areas display the pristine PS surface. The diameter of the etched surface was in the range of 1.5 to 2 mm. Since the thickness of the biofilm varied, some regions were completely free of micro-organisms, whereas some areas were still covered with remnants. Additionally, Fig. 5 shows a sharp boundary between the plasma-affected area, where most of the biofilm was etched, and unaffected biofilm. Similar observation was reported by Koban *et al*. [29]. To get more details on the real extent of the plasma-etched surface, a surface profiling system was applied. Since the texture of the biofilm was too soft for this technique, the surface analysis was carried out on PEEK surfaces; otherwise the biofilm would be scraped off the surface by the diamond needle of the surface profiler. The surface profile of the plasma-treated substrate was measured through the zone of the localized plasma treatment on the polymer (line scan) (see Fig. 6 with inset). Figure 6 displays the surface profile of PEEK exposed to Ar/O_2_ gas discharge for 60, 120, 180, and 300 s, respectively. Note that ‘0’ represents the position of the localized plasma treatment. The surface profiles exhibit that the etching depth increased with prolonged plasma exposure. Furthermore, the most intensive etching process occurred directly on axis of the Ar/O_2_ plasma jet (see also [22]). Hence, the removal of PEEK was mainly restricted to an area of 2 mm in diameter, which corresponds to the finding of the biofilm-etched area in Fig. 5. However, the observed distinct border between unaffected and etched-area after Ar/O_2_ plasma (see Fig. 5) might be due to the localized presence of reactive plasma species. Hence, the distribution of selected optical emission lines of atomic Ar at 750.4 nm and atomic O at 844.6 nm was analyzed along the radial distance which is shown in Fig. 7. Further shown in Fig. 7 is the radial surface profile of PEEK after 180 s Ar/O_2_ plasma exposure to illustrate the correlation between plasma species and surface etching. It can be seen that the etching profile of PEEK proceeds along with the intense optical emission of Ar and O. Hence, the highest intensities of emission lines were directly obtained in the region of the impinging plasma jet. Beyond the gas discharge, the emission intensity declined with increased radial distance. According to the radial measured OES spectra, the surface profile of PEEK showed an enhanced etching effect in the range of the highest emission intensities of Ar and O. Consequently, it can be assumed that the localized etching process is attributed to the presence of reactive species in the impinging plasma effluent, whose effectiveness in surface etching decreases with radial distance. Although OES is a suitable technique to verify excited species, it is not applicable to measure densities of species in the ground state. For this purpose laser-based plasma diagnostic (e.g. photon absorption laser induced fluorescence – TALIF) will be used in future studies to elucidate the influence of O and O_3_ in plasma-assisted etching processes in more detail.

The mechanisms of plasma etching at atmospheric pressure are not yet fully understood, but it is most likely that the main mechanism is based on the chemical process of oxidation which might be described as follows: (i) impinging plasma-emitted reactive species like excited atoms/molecules and radicals result in bond-breaking of molecules, particularly hydrocarbon compounds; (ii) in following reactions two main processes occur simultaneously: modification and etching of plasma-created open bonds on the surface of micro-organisms, leading for instance to the formation of molecular fragments and volatile compounds emanating from the cells. Hence, plasma-exposed micro-organisms exhibit morphological changes, such as reduction in cell size [30], [31], [32] or the appearance of deep etch channels in the cell [33] up to complete cellular destruction [34]. Especially the presence of chemically reactive species, like atomic oxygen and ozone, easily react with these open bonds, which facilitates a faster etching of molecules [22], [33].

Summarizing, the results of this study provide evidence that non-thermal atmospheric pressure plasmas are efficient in etching of 7-day old *Candida albicans* biofilms. Moreover, it was shown that an almost complete elimination of biofilm was achieved by adding molecular oxygen to the argon gas discharge plasma. Hence, Ar/O_2_ plasma efficiently removes biofilms with an etching rate of approximately 33 to 67 nm/s. This etching rate corresponds to rates on previously studied synthetic polymers. The high chemical activity of Ar/O_2_ gas discharge is based on the presence of reactive atomic and molecular radicals interacting with organic materials resulting in etching. These findings may help to modulate plasma processes, which enhance the production of desired reactive species for improved process efficiency. Consequently, the atmospheric pressure plasma jet offers the possibility to eliminate micro-organisms.

## Materials and Methods

### Pre-treatment of the PS wafer

*Candida albicans* biofilms were cultured on polystyrene (PS) wafers (diameter of 10 mm) purchased from Becton Dickinson (Falcon™ Ref 351016). PS wafers were used because of their uniformly flat surfaces which enable a better microscopic imaging and simplify the measurement of the biofilm thickness. Previous cultivation experiments have shown that the formation of *Candida albicans* biofilm on pristine PS surfaces was not homogenous and partly washable by water. Therefore, the PS wafers were amino-functionalized in a low-pressure microwave (2.54 GHz) plasma processing reactor (Plasma-Finish, Schwedt, Germany) using 40 sccm pure ammonia to support the adhesion of *Candida albicans* cells. The PS wafers were exposed for 30 s in the downstream region of the gas discharge plasma. Further information about the reactor as well as the functionalization procedure is described in [35]. The ammonia plasma-treated PS surfaces showed a non-washable dense packed *Candida* biofilm after cultivation.

### Biofilm formation

The strongly biofilm-forming strain *Candida albicans* ATCC 10231 (ATCC = American Type Culture Collection, Rockville, MD, USA) was used [36]. *C. albicans* was suspended into YPD Broth (Yeast Extract Peptone Dextrose, Sigma, Steinheim, Germany). The sterile test objects (PS wafers) were positioned in 24-well microtitre plates (Techno Plastic Products AG, Trasadingen, Switzerland). 1 ml micro-organism suspension (concentration of 10^6^ cells/ml) was added, and incubated aerobically at 37°C. Every 24 h the medium was changed. After 7 days the medium was drawn off and the PS wafers were washed with 0.9% NaCl solution.

### Plasma source and plasma treatment

**Figure 8:**
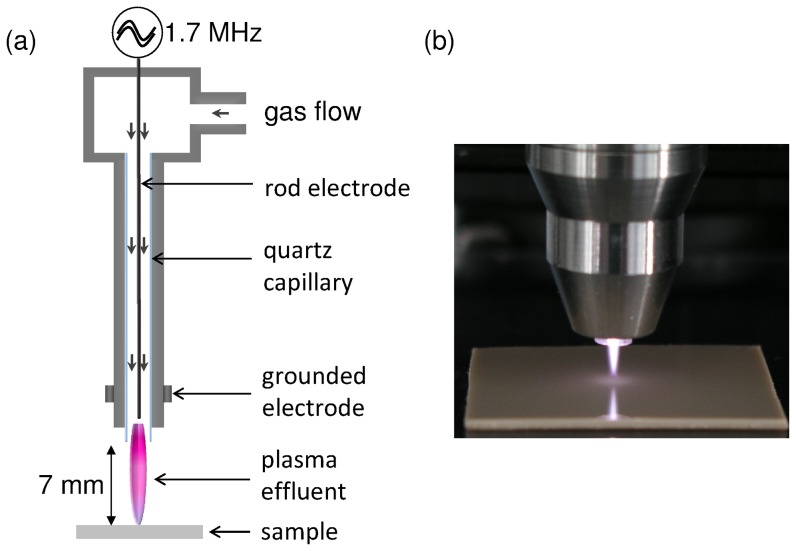
The atmospheric pressure plasma jet (kINPen08, INP Greifswald, Germany). (a) Schematic set-up of the plasma jet which consists of a pin-type electrode centered inside a quartz capillary with an inner diameter of 1.6 mm and an outer diameter of 4 mm. A high-frequency voltage (1.7 MHz, 2–6 kV_PP_) is coupled to the electrode. The overall electric power of the plasma device is 65 W. The plasma jet was positioned perpendicular to the sample surface with a distance of 7 mm between the nozzle outlet and the substrate surface. (b) Photograph of the plasma jet, driven with 5 slm Ar and 0.05 slm O_2_, impinging the PEEK surface.

For the experiments a high-frequency (1.7 MHz, 2–6 kV_pp_) driven atmospheric pressure plasma jet (kINPen08, developed at the INP Greifswald) was applied (see Fig. 8). This plasma device consists of a pin-type electrode mounted in a quartz capillary (inner diameter of 1.6 mm and outer diameter of 4 mm) with an overall electric power of 65 W. The neutral gas temperature was below 80°C under the experimental conditions. A more detailed description of this type of plasma jet, version kINPen09, can be find in [25]. The influence of two different process gases on the removal of biofilms was investigated. In particular, argon (Ar) as feed gas with a gas flow of 5 standard liter per minute (slm) and a gas mixture of argon and oxygen (O_2_) with an admixture of 0.05 slm molecular O_2_ (1% O_2_). Previous studies have shown that the highest etching rate is achieved at small distances between the nozzle-outlet and the substrate surface [22]. Hence, the samples were located at a constant distance of 7 mm from the jet-nozzle. At this working distance the tip of the Ar/O_2_ plasma jet comes in contact with the substrate surface, whereas the Ar gas discharge plasma, with a total length of 12 mm, spreads on the treated surface. The biofilm samples were treated for 60, 120, 180, and 300 s. The control samples were exposed to the gas flow without plasma ignition. Before plasma treatment each sample was washed for several minutes using distilled water and dried by air flow to ensure that the substrate is covered with an adherent, non-washable biofilm.

### Light Microscope

The opaque biofilms were analyzed with a reflected light microscope (Zeiss Axiovert 200 M, Carl Zeiss Jena GmbH, Jena, Germany). Images were taken with a 50 and 200 fold magnification and the biofilm-covered surface area was quantified (200× magnification) with an image analysis program (analySIS® pro V. 3.0, Olympus soft imaging solutions, Münster, Germany). To obtain the biofilm-covered surface area before and after plasma treatment, the surface area of each biofilm, grown on PS, was manually traced and labeled by using analySIS®pro which subsequently calculated the marked surface area. The initial biofilm covered an averaged surface area of 35171±3335 µm^2^ (n = 60, before treatment). To ensure that before and after plasma treatment the same position was microscopically analyzed every PS wafer was marked on the back side.

In order to estimate an approximate etching rate *R* the thickness of the initial biofilm was calculated using a digital optical microscope system. Therefore, a stereoscopic image reproduction with spatial depth using the Zeiss microscope, a CCD-Camera as well as digital electronics for the processing of image data were applied. Data processing proceeds in the following way: an exposure series with constant focus position is taken. The relative movement between the sample plane and sample is carried out in a stepwise manner, where surface configuration of the sample is reconstructed from a set of optical section images to be calculated. Deduced from these images a 3-D image is generated from which the thickness of the biofilm was obtained.

### Statistical analysis

All data presented are means ± standard deviation. For each process gas and treatment time the experiments were repeated five times on five independent biofilm-covered PS wafers (total number of PS wafers: n = 60), whereas for the determination of the biofilm thickness three different positions on the same sample was investigated of at least five selected samples.

### Optical emission spectroscopy (OES)

For the characterization of the plasma phase in the ultraviolet/visible (UV/VIS) spectral range, optical emission spectroscopy was applied, which is a commonly used diagnostic technique to identify excited species (e.g. atoms and molecules) present in the plasma. This method is easy to apply, very sensitive, and most important non-invasive [37]. A dual channel fiber optical spectrometer (Avantes AvaSpec 2048-2-USB2) was used. The channels were linked by an optical fiber to a cosine corrector. A quartz glass was in front of the cosine corrector. The emission lines of the plasma irradiation were measured in the range of 200 to 962 nm. OES spectra were relative calibrated, normalized to the exposure time, and analyzed using the software Spectrum Analyzer [38]. In order to analyze the radial distribution of the plasma species the fiber optics were mounted end-on by a movable holder.

### Polymer treatment and surface profilometry

The depth profile was recorded crossing the centre of the plasma-treated polymer with a stylus surface profiling system (Dektak 3ST, Veeco, USA) with a scan speed of 40 µm/s and a force of the stylus tip on the surface of 10 mg. These investigations were focused on poly(ether ether ketone) (PEEK, purchased from Goodfellow, Germany) because of its very smooth and inflexible surface, being highly suitable for these investigations. According to the treatment of the biofilms, PEEK samples were exposed for 60, 180, 120, and 300 s to Ar/O_2_ plasma (1% O_2_) at a jet-nozzle to substrate distance of 7 mm (see Fig. 8 b).
